# Improving agricultural knowledge management: The *AgTrials* experience

**DOI:** 10.12688/f1000research.11179.2

**Published:** 2017-10-09

**Authors:** Glenn Hyman, Herlin Espinosa, Paola Camargo, David Abreu, Medha Devare, Elizabeth Arnaud, Cheryl Porter, Leroy Mwanzia, Kai Sonder, Sibiry Traore

**Affiliations:** 1 International Center for Tropical Agriculture (CIAT), Cali, Colombia; 2 CGIAR Research Program on Climate Change and Agricultural Food Security (CCAFS), Cali, Colombia; 3 CGIAR Systemwide Management Office, Montpellier, F-34394, France; 4 Bioversity International, Rome, 00054, Italy; 5 University of Florida, Gainesville, FL, 32611, USA; 6 International Maize and Wheat Improvement Center (CIMMYT), Mexico DF, Mexico; 7 International Crops Research Institute for the Semi-Arid Tropics (ICRISAT), Bamako, Mali

**Keywords:** Crop trial, variety, trial site, metadata, CGIAR

## Abstract

*Background: *Opportunities to use data and information to address challenges in international agricultural research and development are expanding rapidly. The use of agricultural trial and evaluation data has enormous potential to improve crops and management practices. However, for a number of reasons, this potential has yet to be realized. This paper reports on the experience of the *AgTrials* initiative, an effort to build an online database of agricultural trials applying principles of interoperability and open access. *Methods: *Our analysis evaluates what worked and what did not work in the development of the *AgTrials* information resource. We analyzed data on our users and their interaction with the platform. We also surveyed our users to gauge their perceptions of the utility of the online database. *Results: *The study revealed barriers to participation and impediments to interaction, opportunities for improving agricultural knowledge management and a large potential for the use of trial and evaluation data. *Conclusions: *Technical and logistical mechanisms for developing interoperable online databases are well advanced.  More effort will be needed to advance organizational and institutional work for these types of databases to realize their potential.

## Introduction

Agricultural research produces thousands of technology evaluations, most of which are crop variety trials. Such trials are carried out in small plots, where researchers can evaluate different plant materials across an entire growing season. Many have been conducted by researchers at the 15 Centers of the CGIAR system, a leader in international agriculture for research and development. National agricultural research institutes, universities, nongovernmental organizations (NGOs), the private sector, and farmers also conduct agronomic and breeding trials in efforts to improve farming system productivity and profitability. There is substantial variability in the quantity of experiments and the quality of data produced among these different actors.

The potential uses of agricultural trial data are enormous. Genotypes can be targeted to the environments where they are most likely to succeed based on their performance in crop trials ( Hyman *et al.*, 2013). Appropriately-targeted cultivars can improve yields substantially ( Annicchiarico *et al.*, 2005; Annicchiarico *et al.*, 2006), particularly when combined with site similarity methods, yielding information on analogous sites ( Jarvis *et al.*, 2014; Jones *et al.*, 2005; Ramírez *et al.*, 2011). Agronomic data can also be used as a benchmark in yield gap studies for what farmers might be able to achieve under improved conditions and management ( Gustafson *et al.*, 2014). Trial data collected across a large geographic extent and over decades can be useful to monitor climate change or the spread of pests and diseases ( Gourdji *et al.*, 2012; Lampe *et al.*, 2014; Lobell *et al.*, 2011), to understand the drivers of technology adoption, to set research and development priorities and to conduct both ex-ante and ex-post impact analysis ( Badu-Apraku *et al.*, 2011; Hyman *et al.*, 2016; Renkow & Byerlee, 2010; Setimela *et al.*, 2005). One of the most obvious uses of agricultural trial data is to calibrate crop models, for a single location or for spatially explicit models covering countries, regions or the entire globe.

Future use of agricultural trial databases will likely be driven by the increased linking of genotype and phenotype to improve selection and use of germplasm, a growing trend driven by advances in molecular biology and site-specific agriculture. These possibilities suggest great potential for the growing “big data” movement in agriculture to use trial data as part of its larger goal to transform the sector. Combination of agronomic data from field trials with genomic data shows promise for developing next generation breeding and selection tools using models ( Hwang *et al.*, 2016). However, the dispersion, lack of organization and inaccessibility of agricultural trial data hinder their use and applicability for resolving problems in agriculture.

In an effort to make datasets of agricultural trials publicly and widely available, the *AgTrials* initiative received startup funds from the Gates Foundation to design and populate an online database, mostly the evaluation of crop varieties. We engaged CGIAR centers, their partners and any others carrying out trials or evaluating agricultural technology, with the aim of populating a database and making the data easily accessible to interested data users. Our strategy was to create a website that represented a network of institutions and researchers. Data providers can control how their data is shared with stakeholders. In some cases, they may only share metadata, requiring interested users to contact the data provider to establish collaboration. The most important element of our strategy was to standardized data enabling users to evaluate multiple trials conducted by different researchers or organizations. The users and providers of data could be breeders and others working on crop improvement, agronomists and agricultural systems specialists looking to improve farming practices or modelers interested in calibrating their models to simulate.

Our project team developed a website and a network of data providers and users. After an initial startup period, the *AgTrials* agricultural data repository ( http://agtrials.org) was further supported and developed by the Climate Change, Agriculture, and Food Security (CCAFS) Collaborative Research Program of CGIAR, in collaboration with a number of national and international partners. *AgTrials* provides access to standardized agronomic trial information for the benefit of future climate change analyses, multi-environment trials and research and development in international agriculture, supporting increased collaboration between countries and institutions across the developing world.

The AgTrials initiative draws on both recent and legacy data. Programs such as CCAFS required recipients of project funds to upload recent data from ongoing research. However, most of the data is from past research and development programs. For example, the two largest sources of legacy data are the International Maize Improvement Network (IMIN) and the International Bean Yield Adaptation Network (IBYAN). These multi-environment trials systems have operated over many years, carrying out trial work under a broad range of environmental conditions ( Reynolds *et al.*, 2012). In other cases, a breeding program shared trials from one or more research stations, trials that were part of their ongoing crop improvement effort. Trials were also shared from fixed-period projects related to particular biotic or abiotic constraints.

One of the *AgTrials* approaches to standardization is to leverage the Crop Ontology Curation Tool ( http://www.cropontology.org/). *AgTrials* includes a dynamic link to the Crop Ontology so that traits or variables measured in trials appear with hyperlinks to their definitions in the Crop Ontology. This capacity allows users to search for a variable measured in any trial in the *AgTrials* database, and combine it with other trials produced by different data providers. For example, two CGIAR Centers, CIAT and IITA, both conduct large numbers of trials on cassava, the former concentrating work in Latin America and Asia, while the latter focuses on sub-Saharan Africa. In a hypothetical example, a researcher working on resistance to green mites may be searching for germplasm tested in both regions by different organizations. The implementation of Crop Ontology in *AgTrials* permits this researcher to evaluate the same cassava green mite severity variables from different data providers, as long as data providers have ensured that their trait names are standardized to the Crop Ontology. *AgTrials* data still needs a great deal of work to ensure standardization, but without standardized terms, the utility of a global trial database is dramatically reduced.

An initial effort to link trial data to crop modeling was developed with researchers of the Agricultural Model Inter-comparison Project (AgMIP; Rosenzweig *et al.*, 2013). Project researchers have used application programming interfaces (APIs) that permit AgMIP systems to view *AgTrials* data and vice versa. AgMIP has also developed a protocol for downloading *AgTrials* data to a suite of crop models ( Porter *et al.*, 2014). The link between agricultural trial data providers and the crop modeling community shows the potential for combining initiatives for analysis of crop improvement potential.

The *AgTrials* initiative also made some efforts to link with other agricultural data initiatives or databases. If we can link the phenotypic data from trials carried out in multiple environments to data held in gene banks, gene expression studies could support the analysis of genotype by environment interactions. The variety names in the *AgTrials* database are also found in gene bank databases. Whereas gene bank data may focus on phenotypic characteristics, the *AgTrials data* is focused on yield or resistance to environmental constraints. We carried out some successful tests to find these links for some of the cassava trials in the database. The link between evaluation trial data and gene bank data could be explored in greater detail with the *Genesys* initiative, a portal linking to germplasm accessions in gene banks around the world ( https://www.genesys-pgr.org/). AgTrials could also interact with the CGIAR’s Integrated Breeding Platform (IBP), an initiative to develop methods and tools for crop improvement ( https://www.integratedbreeding.net/). The efforts described above have been explored, but not yet implemented. There are likely a number of other initiatives where these types of information resources could be integrated.

The developments of the *AgTrials* initiative described above have provided us with an initial experience in the construction of an interoperable agricultural trial database. To date, our experience with the *AgTrials* initiative suggests that research and development advances from using agricultural trial databases will require increased collaboration among and within public and private sectors. The private sector may not share data because their business case depends on not making it widely accessible. Public sector research and development faces a number of obstacles for increased collaboration. Researchers may refuse to make their data available because they have not yet published their findings, their data is not well organized, or they have no incentive to share. Organizational and access issues include the frequent lack of consistent metadata and standards to enable interoperability, resulting in problems of data integration, which is a key requirement to addressing global agricultural challenges.

Thus, our experience to date in the use of agricultural trial data across organizations, countries and continents presents both problems and opportunities. This evaluation assesses the experience of the *AgTrials* initiative in greater depth, reporting on the results of a survey administered via its network of users. Our aim is to consider prospects for developing a larger initiative to promote the development and use of agricultural trial data in the future.

## Methods

The objective of our analysis was to understand what worked and what did not in an effort to develop a global database of trials and evaluations of agricultural technology. Our approach was to review user experience in the *AgTrials* initiative to date, to analyze user interaction and usage data, and to survey users on their experiences. Our analysis includes information from the project website ( www.agtrials.org) from mid-2011 to the end of 2016. We reviewed records from the sign-up information provided by users when registering on the website, including their motivation for participation. Statistics reports provided by Google Analytics were used to assess traffic on the website, along with the record of data downloads. The *AgTrials* website also includes data and statistics on the institutions providing data, the crops for which data is available, the location of trials and the participation of institutions involved in the initiative.

The project team administered a survey in August 2016 to evaluate the perspective of the *AgTrials* user base (survey results available from https://en.surveymonkey.net/results/SM-YLGDFPQG/) and survey data accompanies this article ( Dataset 1; Hyman *et al.*, 2017). As an incentive to participate in the survey, users were offered the possibility to enter a draw for a smartphone, an incentive reflected in the 44% response rate of users who received an invitation. 146 of 326 registered and active users took part in the survey; 19 of the survey replies were not fully complete. The survey covered topics on incentives and motivations for providing data, use of data, the potential for a global repository of trial information, and other topics. A copy of the survey questions can be found in Supplementary File 1.

## Results

### Registered users and the website

**Figure 1.**
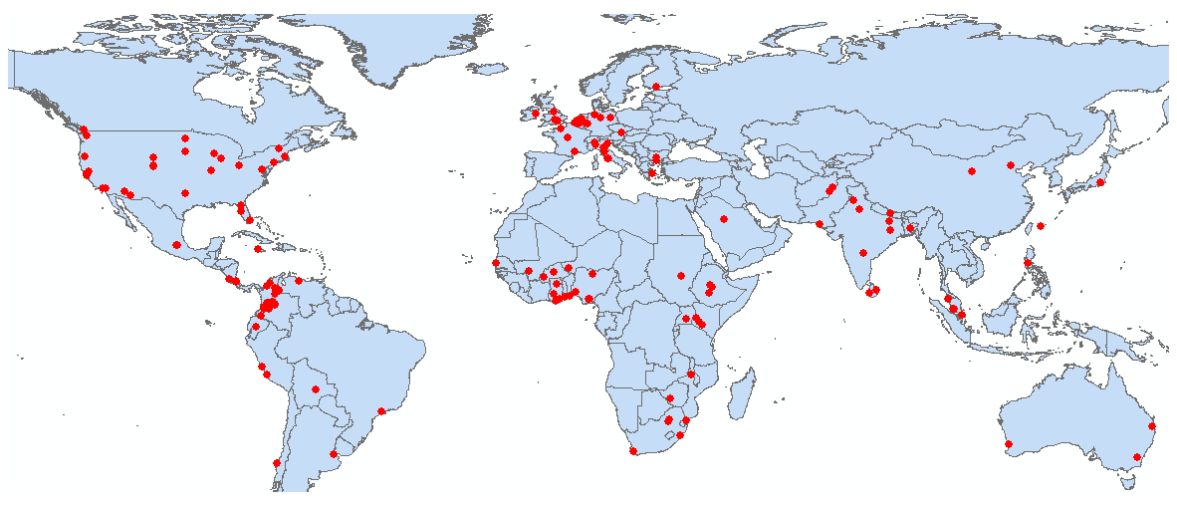
Geographic distribution of registered users to the *AgTrials* website.

More than 400 people have registered on the *AgTrials* website, providing their contact information and their motivation for participation ( Figure 1). However, 326 of the registered users are considered active, having returned to the site after registration. Between the middle of 2011 and July 2016, the website had 25,648 visits, 64% of these being return visits. Most of the visitors were from Colombia, reflecting the program’s outreach from the International Center for Tropical Agriculture (CIAT), which hosts the initiative and is based in Cali, Colombia. Large numbers of visitors were also registered from the United States, Nigeria, Kenya and the Netherlands. Our data users downloaded 1,531 datasets during the analysis period.

***User motivations*.** The stated motivations for joining the initiative varied across the more than 400 users from across the world ( Figure 1), with the following being the most common reasons to look for data in *AgTrials*: for validating crop models; for studies on genotype by environment interaction; for acquiring daily weather or soil information; or for use in the context of studies on climate change. Many users were students working on a thesis project. The initiative came to the attention of at least two hackathons – events where coders and developers use API to bring data into their own applications. Some registered users of *AgTrials* were professionals working on aspects of open data initiatives, interested in data curation, metadata, and how the initiative was set up. Some of these were working with other networks that use agricultural evaluation data, such as the AgMIP ( http://www.agmip.org/) and iPlant (rebranded as CyVerse, with the URL http://www.cyverse.org/) initiatives. A few research and development donors and their beneficiaries joined the initiative to share and verify evaluations resulting from their projects.

**Figure 2.**
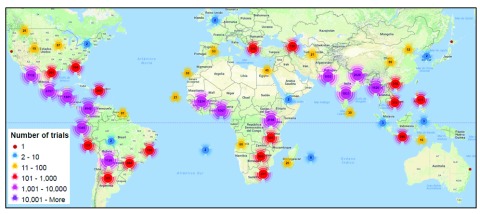
Geographic distribution of the number of trials in the *AgTrials* database.

**Figure 3.**
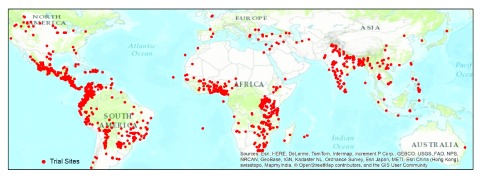
Geographic distributions of trial sites across the world that have at least one trial for which there is data in the *AgTrials* database.

***Trial contributions by users*.** Users of the *AgTrials* platform contributed over 35,000 records of trial information from locations across the world ( Figure 2). Approximately 85 percent of the trial records only include metadata, obligating those interested in the data to directly contact the information provider. The large majority of trials in the database were maize (29,461), followed by common bean (1881), cassava (1751), rice (411) and forages (405) to round out the top five. The countries with the largest number of trials in *AgTrials* were Mexico, India, Colombia, Guatemala and Ethiopia – making up over half the trials in the database. Cotaxtla and Tlaltizapan in Mexico, Palmira in Colombia and Las Vegas, Guatemala were among four trial sites that contributed more than 500 trials to the database. Other sites that were large contributors of trials include Agua Fria, Mexico, Hyderabad and Bangalore in India, Nioro in Senegal and Bako in Ethiopia. The top research centers contributing data were the International Maize and Wheat Improvement Center (CIMMYT), the International Center for Tropical Agriculture (CIAT), the International Institute of Tropical Agriculture (IITA) and the International Crop Research Institute for the Semiarid Tropics (ICRISAT); all centers of the CGIAR network. The database contains trials from 2,553 sites ( Figure 3), most often national partners and collaborators of the CGIAR Centers, which generate most of the data.

### *AgTrials* user survey

***User profiles*.** The *AgTrials* user survey is based on responses from 146 registered users of the platform who provided information. Nearly 40% of these were from CGIAR Centers, 20% from universities, 14% from government agencies, 6% from NGOs and 8% from the private sector. More than 43% of those surveyed had actually used the data in the context of their original motivation for registering for the platform. For these users, the data was useful for crop modeling and evaluation, genotype by environment interaction studies, phenological studies, as reference data, for data curation of their own databases, for checking weather data, for responding to data requests and for research on data repositories.

***AgTrials-relevant data in institutional repositories*.** The survey asked users how many trials their institution holds that can potentially be part of a global trial repository. 53% of respondents (equivalent to 77 people) indicated that their institution holds between zero and 100 trials that could potentially be part of a database. A total of 16 respondents (11%) suggested they could provide between 100 and 250 trials. Another 15 respondents (~10%) believed that their institutions can provide between 250 and 50,000 trials. Finally, four respondents indicated that their institutions could provide more than 50,000 trials. When asked to comment on the number of trials that might be part of a repository, there was a range of replies. Several users said that their organization did not have permission to publish trials of farmer partners. Many of the respondents simply did not know, primarily because they worked in large organizations and didn’t have numbers for trials carried out in other departments or other research locations.

***Barriers to contributing data to AgTrials*.** The survey also asked users about barriers that discouraged them from contributing data to the *AgTrials* platform ( Table 1). Of the 112 respondents to this question, the most common answer given by ~34% of the respondents revolved around their belief that their data was not sufficiently organized for public sharing. 28% indicated that they needed more funding and resources to help them organize and upload data. 27% of the respondents believed that they or their institution simply did not have data to contribute. Another important reason for not wanting to contribute data, offered by 22% of respondents, was that it had not yet been published, and they did not want to share it until they had a chance to publish. Nearly 17% of respondents said that their data was published on another platform and that they did not want to duplicate efforts. Some respondents (13%) were discouraged from contributing data because they do not know how to upload or make it available. Over 12% of respondents considered that the policies or institutional culture of their institutions discouraged or forbade them from making data available. In about 10% of the responses, users said that donors or partners had asked that data not be made open access. Nearly 10% of the respondents indicated that they did not like the technical and design aspects of the *AgTrials* platform.

**Table 1.** Number of users citing different barriers to data sharing by the individual user or their institution.

What are the barriers that discourage you or your institution from contributing data to the AgTrials platform? (check all that apply)		
Answer Options	Response Count	Response Percent
Our data are not sufficiently organized/clean for public sharing	39	34.8%
I need to receive funding to help me organize, document and upload the data	32	28.6%
I do not myself (nor does my institution) have data to contribute	31	27.7%
I have not yet published my research and do not want to make the data available until I have published	25	22.3%
Other (please specify)	23	20.5%
Our data are published in a different platform and I do not want to duplicate effort	19	17.0%
I don’t know how to upload and contribute data	15	13.4%
Policies or institutional culture of my institution either discourage or forbid that I make the data available	14	12.5%
Donors or partners have asked that the data are not open access	12	10.7%
I do not like certain aspects of the AgTrials platform, for example the data format or the submission process	11	9.8%

***Incentives to encourage contributions to AgTrials*.** The survey asked users what some incentives might be that would motivate them or their institution to contribute data to the platform ( Table 2). Over 60 respondents, equivalent to 53%, indicated that the possibility that their data contribution could be cited and acknowledged would be a motivating factor for contributing to the platform. An equal number of users specified that they would be motivated by the possibility that their data could be combined with other datasets, dynamically linked to other platforms to facilitate meta-analyses or linked to larger studies. More than 44% of respondents cited the value of organizing their data in an application specifically designed for managing and sharing agricultural trial data. More than 43% of respondents suggested that they recognize the value of their data and they didn’t want it to be lost, but rather remain available and useful to others. Other incentives and motivating factors for contributing data included being able to comply with their organization or donor’s data policy (38%), if they were to receive funding to organize, document and upload the data (29%) and whether recognition of their data contribution could support their institutional or individual performance evaluation (25%).

**Table 2.** Incentives cited by survey respondents that would motivate individual users or their institutions to contribute data to the *AgTrials* platform.

What are some incentives that would motivate you or your institution to contribute data to the AgTrials platform? (check all that apply)		
Answer Options	Response Count	Response Percent
The possibility that my data contribution can be cited and acknowledged	60	53.6%
Being able to organize my data in an application specifically designed for managing and sharing agricultural trial data	50	44.6%
I need to receive funding to help me organize, document and upload the data	33	29.5%
Recognition of my data contribution in my performance evaluation	28	25.0%
Being able to comply with my center's or donor’s data policy	43	38.4%
My data could be combined with other datasets or dynamically linked to other data platforms to allow meta-analyses or to contribute to larger research studies	60	53.6%
I recognize the value of my data and I don’t want it to be lost. It should continue to be available and useful to others	49	43.8%
Other (please specify)	19	17.0%

Surveyed users were asked whether they would be willing to provide both metadata and the full dataset, only metadata without the data, or neither data nor metadata. Almost 67% of respondents agreed that they were willing to provide both metadata and the full dataset. More than 27% would be willing to provide metadata only, giving their contact information in order to establish subsequent communication with interested users of their data. In total, 5% of respondents would be unwilling to provide either data or metadata.

***Data sources for a global repository*.** The survey asked users what sources of data should be collected in a global repository. Users indicated that data collected from CGIAR centers (89% of those surveyed) and national agricultural researchers systems (86%) should be included. 81% of those surveyed suggested that trial data from farmer fields carried out through farmer participatory research should be included. Only 59% of those surveyed indicated that data from seed companies or agroindustry should be included in a global trial repository. Users also cited the inclusion of data from agronomic trials that test management practices, environmental and remotely sensed data related to trials and data from breeder trials focus on selecting varieties that perform well.

***Applications of a global agricultural repository*.** Respondents to the survey also provided information on key applications of an international database of agricultural trials. Several respondents mentioned the use of data for crop modeling, site similarity and other spatial analysis. These applications would evaluate the performance of crop varieties in different places and then estimate where else they might do well, suggesting where a variety release program might focus its activities. Applications of the dataset might examine crop pests and diseases and how their impacts vary with location. Other suggestions considered that the data would be useful in “big data” combinations and analyses of different datasets. For example, one respondent suggested that GPS coordinates from the trials could be used to acquire remote-sensing imagery for each plot, enabling the use of the acquired imagery for phenotyping. Respondents mentioned the possibility of using pedigree information to link genotype to phenotype in order to understand molecular level dynamics in different environmental settings. Several respondents mentioned the possibility of combining trial data with household surveys to evaluate possibilities for variety adoption. Another group of applications were suggested around priority setting and impact assessment, using crop performance data as a measure of the level of ex-ante impact.

***General user reflections*.** Finally, respondents were asked to reflect on how this type of international trial database initiative could improve, and what direction it might take in the future. Several respondents mentioned the need for technical improvements in the structure of the database and the user interface. A few respondents said that it simply needed more data. Others mentioned the need for capacity building among users in order to be able to use the data more effectively. Many respondents indicated that the combination of this data with other existing datasets needed to be exploited to realize the full potential of the information resource. Several respondents said that the information resource leads to better integration of open data concepts, such as interoperability, metadata and data discovery and sharing. Many respondents mentioned the need for a stronger and more organized institutional arrangement to develop and use agricultural trial information.

Dataset 1. Anonymous individual responses and survey questions http://dx.doi.org/10.5256/f1000research.11179.d155255
Copyright: © 2017 Hyman G et al. 2017 Data associated with the article are available under the terms of the Creative Commons Zero "No rights reserved" data waiver (CC0 1.0 Public domain dedication).

## Discussion

Our experience to date in developing an agricultural trial database and the responses to our user survey suggest a number of key topics that need to be considered in the future development of this or similar initiatives. These topics concern the quality and quantity of data, improvements needed in the database and website, possible combinations of this information resource with other information initiatives, open data and data infrastructure issues and institutional arrangements to make this kind of effort succeed.

One interesting question surrounding this initiative is what its scope might be in terms of the quantity of data that might be included. Currently there are 35,000 trials in the database, mostly reflecting a large number of CIMMYT maize trials, plus a smaller collection of other crops. Respondents who said that their organization could potentially contribute more than 50,000 trials were from CGIAR centers. About seven of the CGIAR centers have major breeding programs, and if each of these could contribute 50,000 trials, the total would be 350,000 trials. Many of these CGIAR-reported trials are carried out by national agricultural research institutes and are simply part of larger CGIAR databases. There may be a number of other international organizations that participate in the initiative and could perhaps contribute more than 100,000 trials. There may be no way to know how many trials are carried out by agricultural colleges, universities, NGOs and others around the world. But overall we can speculate that the potential size of this database could be between 500,000 to 1 million agricultural trials. If a database initiative could reach those kinds of numbers, it is easy to see the potential of this information for all kinds of research and development.

Another set of issues related to agricultural trial data concerns the quality of the data and the database itself. It is notable that respondents to the survey said one of the greatest barriers to including data was that their information was not well organized and documented. Our experience has been that data curation efforts have lacked the resources needed to ensure data quality. Data curation is not one of the most exciting tasks, but it is critical for subsequent use of trial result data. Legacy data very often suffers from data quality problems. Therefore, data providers need to use best practices at the moment that data is recorded, as well as curate legacy data to overcome any deficiencies in the original collection of trial information.

Any global trial data and information resource must pay considerable attention to the details of developing quality data that is well documented and an information system that facilitates ease of use in providing or acquiring data. For example, some *AgTrials* users pointed to the lack of detailed information on experimental design of the trials. Crop modelers typically want as much phenotypic data as possible, but also detailed data on the weather during the trial and the soil conditions of the trial site. For legacy data, it is perhaps unrealistic to expect huge numbers of trials with the full data that a crop modeler might want. But at the very least, data documentation and search mechanisms need to give users a clear picture of the data available in any given trial or set of trials. Therefore in the future of this or similar agricultural trial data resources, developers must make the needed investment to ensure that the data and the interface with the data meet minimum standards for documentation and ease of use.

The open data movement in agriculture is a trend that will surely affect the development of data and information resources like *AgTrials*. The Integrated Breeding Platform is one such initiative ( https://www.integratedbreeding.net/). This CGIAR–supported program is aimed at developing methods and tools for public sector breeding. The initiative does make some data available, although providing data is not a key focus of the program. This type of program could work interoperably with *AgTrials* providing public sector breeders an outlet for sharing their information. The AgMIP initiative mentioned above also makes available trial data ( https://data.agmip.org/cropsitedb). The datasets could be from variety trials, but could also be field experiments with multiple factors such as N, water, temperature, CO2, cultivar, etc. The AgMIP datasets are associated with field observations for management and production. For example, even the simplest farm survey data will include lat-long, crop, recorded planting date, fertilizer applied, harvest date, and some estimation of final yield. Field experiments include much richer recordings of all management activities in the field and measurements of crop, soil, and weather variables. Some AgMIP datasets also include simulated results, in addition to the field records. Other such initiatives include the USDA’s Long Term Agro-ecological Research program and an Australian initiative called Online Farm Trials ( https://www.farmtrials.com.au/). The former initiative does not yet provide open access data. Both of these initiatives are focused on their respective countries. European researchers have proposed a pan-European trial data initiative, but it has not yet been implemented ( Stützel *et al.*, 2016). All of these initiatives point to a growing realization that agricultural data integration can be a very powerful way towards crop improvement ( Reynolds *et al.*, 2012; Reynolds *et al.*, 2017).

The CGIAR and many of its centers have recently developed new data policies oriented around data sharing and open access. These policies may motivate producers of trial data to participate in *AgTrials* or similar initiatives as a way to verify their work and share data with stakeholders. However, it will be necessary to emphasize the importance of standardized data initiatives that allow published data to be interoperable. For example, simply uploading data to a system, such as Dataverse, in which providers are not obligated to standardize data, would leave us with datasets that cannot be combined or studied together without a substantial effort by users. *AgTrials* uses the Crop Ontology initiative as the basis for standardizing data and making it interoperable, further development of which is crucial for supporting open trial data ( Matteis *et al.*, 2013). The open data movement’s emphasis on metadata is another development that could lead to better use of agricultural trial data. As the *AgTrials* data resource is adopting the CGIAR metadata schema, new opportunities for data discovery will likely become more apparent. Open data will also promote principles such as clear designation of intellectual property and digital identifiers for interoperability and proper citation, developments that could substantially improve the use of agricultural trial data.

Finally, the survey and our experience in developing an agricultural trial database suggests the difficulties in developing a network of data providers and users. Private sector actors, such as large seed and agricultural input companies, have very well developed trial and phenotyping databases. They have a much more direct line between the data and its impact because understanding their trials and using the data directly affects their profits. As private sector companies tend to be vertically integrated hierarchical organizations, they can enforce discipline among their employees in developing and using agricultural trial data. Public sector efforts, on the other hand, depend on a networking model to organize themselves. They must all agree and be motivated to contribute to a data initiative. There are few negative consequences if they do not pursue an open data policy towards their trial data. Given the international agricultural research environment we are working in, public efforts to build agricultural trial information resources need a combination of carrots and sticks, incentives to participate and disincentives to go it alone. Developing these motivations for participation is particularly difficult considering the large number of stakeholders that would need to be brought together for a global trial data initiative.

## Conclusions

This evaluation considered the development of a global agricultural trial database that can be established and used by a large number of stakeholders interested in crop improvement. Our experience in the initiative to date has shown that this type of effort has great potential for a number of applications. There is very likely a large number of agricultural trials that could be part of a global database. An initiative of this type requires development of well documented data and systems that facilitate ease-of-use. Barriers and incentives to participate could be addressed using a carrot and stick approach, where data providers and users work within an enabling environment to advance the initiative. Changes in practice are necessary for documenting and providing trial data as it is collected from the field or greenhouse. Data providers need to apply best practices and open data principles at the outset of their data collection programs. Legacy data will need increased curation, an effort that is not trivial and requires substantial resources.

A future global agricultural trial data initiative will have to address the need for actors in the public sector to organize themselves around the goals of the effort. International and national institutions would need a strong commitment to participate. Donors to research and development projects would also need to commit themselves to requiring participation from the trial work they fund. The growing open data movement might provide one element of the enabling environment for such an initiative to be successful. The institutional and organizational barriers to creating a global trial information resource are much greater than any technical obstacles.

## Ethics statement

We invited registered and active *AgTrials* users to participate in the survey reported in this paper, guaranteeing that their responses to the questionnaire will remain anonymous. Only the survey administrator was given access to the identity of the respondents, in the case that we wanted to follow up on certain questions. After reviewing survey responses, we determined that there was no need to follow up on questions.

## Data availability

The data referenced by this article are under copyright with the following copyright statement: Copyright: © 2017 Hyman G et al.

Data associated with the article are available under the terms of the Creative Commons Zero "No rights reserved" data waiver (CC0 1.0 Public domain dedication). 

The survey data that was the basis of this paper is available on Survey Monkey at the following URL: https://en.surveymonkey.net/results/SM-YLGDFPQG/. Readers are also encouraged to visit the *AgTrials* website where metadata and data on agricultural trials can be accessed ( www.agtrials.org).

Dataset 1: Anonymous individual responses and survey questions. doi, 10.5256/f1000research.11179.d155255 ( Hyman *et al.*, 2017).

## Supplementary material

Supplementary File 1: Survey questions.
