# Supplementary material for: Improving agricultural knowledge management: The AgTrials experience
Source: F1000Res. 2017 Oct 9;6:317. Originally published 2017 Mar 24. [Version 2] doi: 10.12688/f1000research.11179.2 (PMC5443339; doi:10.12688/f1000research.11179.2)
Supplement: Supplementary file 2 [file f1000research-6-13903-s0001.tgz › 912bedb9-15fc-409d-8073-e02440be1aee.pdf]

## Welcome to AgTrials Consultation

**Several years ago a small group of people in the CGIAR put together a global agricultural trial repository and database called AgTrials. The objective of this information resource is to motivate data sharing that will ultimately lead to improved selection and use of crop varieties and technologies across environments. The initiative has put together standardized information on more than 35,000 agricultural trials. CGIAR researchers are now considering the future of the initiative.**

**As a registered user of AgTrials, we are asking for your help in our evaluation of the initiative. We developed this survey to collect information from AgTrials data providers and users, and others as well. Your responses will help us evaluate the future of this database or a similar initiative to make use of data sets on the performance of crop varieties or technologies. The survey should only take 20 minutes to complete, and we would be very grateful if you would take the time to provide your perspective and information. All responses will remain confidential. All respondents will maintain anonymity.**

**In early September we will schedule an online session to share and discuss the results of the survey. We will keep you informed on the day and time of the session to report and discuss results.**

**Respondents completing the entire survey before Wednesday August 31st will be automatically entered into a drawing to win one of 10 Google Nexus smart phones. Winners will be selected by a random draw *(our targeted survey respondents are 325 AgTrials users, so if half of these fill out the survey, you would have about a 6% chance to win the phone).***

* 1. Contact information

First Name

Last Name

Email address

* 2. Type of User:

- CGIAR
- Government
- NGO/NFP
- University
- Private
- Other (please specify)

## Survey

* 3. Have you used the AgTrials information resource and database? Did you use it in the context of your original motivation to register for the platform?

- ◯ Yes
- ◯ No

If YES, please list your uses of the data and provide references if available. If NO, please explain why you did not use the AgTrials platform:

* 4. Given that a trial is one crop or technology, for one growing season at one location, how many trials might you or your institution hold that could potentially be part of a global trial repository? *(Use drop down menu)*

Please comment if appropriate:

* 5. What are the barriers that discourage you or your institution from contributing data to the AgTrials platform? (check all that apply)

- [ ] I do not myself (nor does my institution) have data to contribute
- [ ] I do not like certain aspects of the AgTrials platform, for example the data format or the submission process
- [ ] I have not yet published my research and do not want to make the data available until I have published
- [ ] Donors or partners have asked that the data are not open access
- [ ] Policies or institutional culture of my institution either discourage or forbid that I make the data available
- [ ] Our data are published in a different platform and I do not want to duplicate effort
- [ ] Our data are not sufficiently organized/clean for public sharing
- [ ] I need to receive funding to help me organize, document and upload the data
- [ ] I don't know how to upload and contribute data
- [ ] Other (please specify)

* 6. What are some incentives that would motivate you or your institution to contribute data to the AgTrials platform? (check all that apply)

- [ ] The possibility that my data contribution can be cited and acknowledged
- [ ] Being able to organize my data in an application specifically designed for managing and sharing agricultural trial data
- [ ] I need to receive funding to help me organize, document and upload the data
- [ ] Recognition of my data contribution in my performance evaluation
- [ ] Being able to comply with my center's or donor's data policy
- [ ] My data could be combined with other datasets or dynamically linked to other data platforms to allow meta-analyses or to contribute to larger research studies
- [ ] I recognize the value of my data and I don’t want it to be lost. It should continue to be available and useful to others
- [ ] Other (please specify)

* 7. With respect to metadata -- such as date of trial, varieties tested, traits measured, data provider contact and other information about the trial -- and data, check the box below that fit with your perspective:

**Use of metadata and data**

- ◯ I would be an interested user of metadata, even if I don't have immediate access to the data
- ◯ I would only be interested in the data resources if I could get **BOTH** metadata and the data
- ◯ Other (please specify)

* 8. With respect to metadata -- such as date of trial, varieties tested, traits measured, data provider contact and other information about the trial -- and data, check the box below that fit with your perspective:

**Provision of metadata and data**

- ◯ I would be a willing provider of metadata only (without the data), giving my contact information to establish how to share the data through subsequent communication with interested users
- ◯ I would be willing to provide both metadata and the full dataset
- ◯ I’m not willing to provide neither metadata nor datasets

* 9. Would linking large databases of genetic information held by genebanks and crop improvement programs to phenotyping data from AgTrials or similar databases be useful for studying genotype-by-environment interactions?

◯ Not useful ◯ Slightly useful ◯ Moderately useful ◯ Useful ◯ Very useful ◯ N/A

* 10. In your work, could agricultural trial data such as that available in AgTrials -- with its advantages and limitations -- be used for improving and calibrating crop models?

◯ Could not be used ◯ Could slightly be used ◯ Could moderately be used ◯ Could be used
◯ Could definitely be used ◯ I don't know

* 11. What types of agricultural trial data should be collected in a global repository? (Check all that apply)

- [ ] Data from CGIAR center trials
- [ ] Trial data from National Agricultural Research System (NARS) partners
- [ ] Data from seed companies or agroindustry
- [ ] Trial data from farmer fields carried out through farmer participatory research
- [ ] Data from breeders' trials focused on selecting varieties that perform well
- [ ] Data from agronomic trials testing management practices as well as variety performance
- [ ] Environmental data from other data providers or remotely sensed data
- [ ] Other (please specify)

* 12. Agricultural researchers have conducted and continue to carry out field trials across the world, trials that tell us about the performance of a crop variety or management practice. How useful do you find this information?

○ Not useful ○ Slightly useful ○ Moderately useful ○ Useful ○ Very useful ○ I don't know

* 13. Some applications of a trial data respository might include (1) combining data with genomic data for development of gene-based models, (2) crop model testing, validation and parameterization, (3) meta-analyses on crop suitability or management optimization, (3) variety selection, (4) genomic selection using models, among others. Could you please list up to 3 other innovative uses of agricultural trial data? *(Write "N/A" if you have no suggestions for data uses)*

* 14. AgTrials is ending a phase of work. Do you think that this type of initiative – one that establishes a global trial repository using standarized terminology (semantics/crop ontologies) – should be continued in the future?

- ◯ Yes
- ◯ No

Please add appropriate comments:

* 15. If you would like to make any other reflections, comments or suggestions about AgTrials or similar initiatives to share crop performance or management practice data, please do so here. *(If not, indicate that you have "no comment").*

**Thank you!**

We will be contacting you shortly with information on the results of the survey and the date and time of an online discussion of the results
