# Supplementary material for: Improving agricultural knowledge management: The AgTrials experience
Source: F1000Res. 2017 Oct 9;6:317. Originally published 2017 Mar 24. [Version 2] doi: 10.12688/f1000research.11179.2 (PMC5443339; doi:10.12688/f1000research.11179.2)
Supplement: Peer review history

# Peer review: Referee response for version 1

**DOI:** 10.5256/f1000research.12062.r22090

**Published:** 2017-05-22

**Authors:** Ruth Bastow

**Affiliations:** 1 Global Plant Council, London, UK

This paper looks at the use of data and data submissions to the AgTrials initiative. To assess what was productive and effective in this initiative and what could be improved if the initiative was developed further. The survey of users, data collection, data analysis and conclusions are clearly described and understandable.

 It would be helpful if the paper had provided a clearer introduction to the AgTrials project perhaps with some examples of data that AgTrials holds. It would also be useful to understand what similar projects or initiatives are being undertaken elsewhere in the world and how they have dealt with the issues raised in the paper. For example the paper notes that 17% of respondents published their data on a different platform.

 As funding bodies, donors and governments push for greater open access/open data the important issues raised in this paper (at both the technical and sociological levels) will need to be dealt with and most importantly coordinated at the international level to help ensure the effective use of agricultural trial data in improving agricultural and agricultural practices.

I have read this submission. I believe that I have an appropriate level of expertise to confirm that it is of an acceptable scientific standard.

# Peer review: Referee response for version 1

**DOI:** 10.5256/f1000research.12062.r21255

**Published:** 2017-04-10

**Authors:** Anne-Françoise Adam-Blondon

**Affiliations:** 1 Research Unit in Genomics-Info (URGI) UR1164, French National Institute for Agricultural Research (INRA), University of Paris-Saclay, Versailles, France

The title is appropriate to the content of the paper, that describes the result of a survey of the users of an open database for crop trials.

 The aim of the survey was to understand globally the usefulness of such a database and precisely the problems encountered by users for contributing to it. The survey was performed with an online, easy tool and the questions are mostly targeted to understand the bottleneck for getting new data into the information system.

 The introduction lacks a bit the strategy of the AgTrial initiative, its positioning in relation with other similar initiatives (at least BMS or others that are developed in the CGIARs): who are the targeted users more precisely. I understand between the lines that they are more agronomists than breeders, although this is not completely sure. The choices in terms of standardization should be also a bit more detailed, with its efforts and gaps: the crop dictionary is used for the traits description but what about the genetic material, which is also a very important object for linking genotypic information for breeders and geneticists?

 The results of the survey are fully provided and interesting, pointing again the need to incentivize and support data curation and data management but also to a better recognition of data ownership to get more data in such central repositories. However, when looking more in detail to the last questions about the usefulness of AgTrial, the question of the links with other initiatives (e.g. BMS, CG Big data) is also strongly argued both to facilitate the submission or the harvest of properly formatted (and standardized?) data and to link data with other data. This should be more clearly discussed as I think this is also a critical issue for the users: when multiple initiative do exist, how do they complement or link between each others?

 The bottlenecks might also be linked to the perception of the usefulness of the data once open in such database. This usefulness is very dependent on the type of standardization (again, the correct identification of both traits and genotypes is crucial for breeders before anything else, while modelers have other priorities) but also on the real accessibility to the data (which may depend on the community you belong to: for instance I was not able to access to the data I searched for).

 The discussion of paper should better address the positioning of AgTrial regarding these two issues (link with other similar initiatives and future possible directions for improvement of the information systems that would enhance usefulness of AgTrial) in different scenarios in terms of users/submitters targeted: really open data for agronomists, really open data for breeders, CGIARs/community data more or less open to the rest of the world for agronomists, same for breeders. The priorities might be a bit different.

I have read this submission. I believe that I have an appropriate level of expertise to confirm that it is of an acceptable scientific standard.
